# The diverse interaction of metabolism, immune response, and viral pathogens

**DOI:** 10.3389/fimmu.2025.1619926

**Published:** 2025-07-29

**Authors:** Toshio Kanno, Keisuke Miyako, Yusuke Endo

**Affiliations:** Department of Frontier Research and Development, Laboratory of Medical Omics Research, Kazusa DNA Research Institute, Chiba, Japan

**Keywords:** lipid metabolism, virus infections, T cells, cGAS-STING, SCD2, immunometabolism, pathogen nucleotide sensor, virus lipid

## Abstract

During viral infections, both innate and adaptive immune responses are activated to establish host defense mechanisms. In innate immunity, the STING and MAVS pathways, which recognize viral genomes, play a central role in inducing type I interferons (IFN-I), a group of antiviral cytokines. Concurrently, adaptive immune responses, particularly those mediated by T cells, contribute to viral clearance and the establishment of immune memory through the recognition of viral antigens. Recently, numerous studies have highlighted the impact of alterations in lipid metabolism on host immune cells during viral infections. Because viruses lack the ability to synthesize their own lipid membranes, they rely on host lipid metabolic pathways to support their replication. In addition, IFN-I signaling has been shown to suppress the expression of lipid metabolic genes and promote the generation of antiviral lipids. Furthermore, following viral infection, both innate and adaptive immune cells rewire various metabolic pathways, including lipid metabolism, glycolysis, the tricarboxylic acid cycle, and amino acid metabolism, to mount effective antiviral responses. This review focuses on recent advances in our understanding of lipid metabolic reprogramming during viral infection at both the cellular and systemic levels, and how such metabolic changes shape and regulate immune responses.

## Introduction

T cells play a central role in adaptive immunity by recognizing foreign antigens through the T-cell antigen receptor (TCR). Naïve CD4^+^ T cells can differentiate following induction by lineage-specifying cytokines into functionally distinct subsets, including Th1, Th2, Th17, and regulatory T cells (Tregs). Those effector CD4^+^ T cells contribute to various immune responses, such as antiviral defense, allergic reactions, and autoimmunity. CD8^+^ T cells also play an important role in the immune response to intracellular pathogens and cancers. In recent years, growing evidence has highlighted that TCR activation induces changes in the expression of genes involved in the cellular metabolism. Furthermore, a paradigm shift in immunometabolism emphasizes that metabolites are not merely building blocks for biosynthesis or substrates for energy generation; they also directly or indirectly interact with major signaling hubs in T cells (1, 2). For instance, enhanced glycolysis boosts the pathogen-eliminating capacity of both CD4^+^ and CD8^+^ T cells by promoting IFNγ production (3–5). Amino acid metabolism supports the generation of antiviral Th1 cells, a subset of CD4^+^ T cells (2, 6). In particular, accumulating evidence has shown that lipid metabolism is crucial for the generation, regulation, and maintenance of effector Th cell subsets. ACC1, a rate-limiting enzyme of fatty acid biosynthesis, has a pivotal role in the differentiation of Th2 and Th17 cells, which is responsible for the pathogenesis of allergic inflammation or auto-inflammatory disease, respectively (7, 8). Tissue Treg homeostasis heavily relies on the mitochondrial fitness in an Acyl-CoA synthetase *Acsbg1*-dependent manner (9). In addition to regulating effector T cells, metabolic pathways are also essential for the generation of memory CD4^+^ and CD8^+^ T cells, thereby contributing to the establishment of long-term immune memory (10, 11). Thus, cellular metabolism appears to play an essential role in modulating a wide variety of adaptive immune responses.

With the advance of immunometabolism research, it has become clear that lipid metabolism plays an important role in viral infections. Type I interferon (IFN-I) contributes to antiviral protection through the upregulation of antiviral IFN-stimulated genes (ISGs). Accumulating evidence indicates that IFN-I also changes cellular metabolism to support the full activation of antiviral responses and the production of antiviral metabolites (12–14). For example, IFN-I signaling enhances fatty acid oxidation (FAO) and oxidative phosphorylation (OXPHOS), which are essential for the regulation of plasmacytoid dendritic cells (pDCs). In addition, IFNβ treatment also enhances the generation of the antiviral lipid 25-hydroxycholesterol (25-HC) in macrophages (12, 13). While the IFN-I response shapes cellular lipid metabolism, metabolic alterations, in turn, can modulate IFN-I signaling. The interaction between viral infection and lipid metabolism also impacts the acquired immune system. In fact, mono-unsaturated fatty acids (MUFAs) metabolism decreases in T cells upon infection, which activates type I IFN and contributes to the antiviral response (14). On the other hand, in macrophages, fluctuations in cholesterol metabolism prime IFN-I signaling (15). While IFN-I and lipid metabolism act in concert to regulate viral infection, viral infections also induce host lipid metabolic reprogramming, which is critical for supporting efficient replication (16, 17).

In this review, we begin by providing an overview of the immunometabolism of CD4^+^ and CD8^+^ T cells. Subsequently, we focus on the significance of IFN-I in metabolic processes during viral infections and summarize the relationship between cellular lipid metabolism and viral infections. Furthermore, we aim to offer a comprehensive examination of the current knowledge of lipid metabolism in controlling the viral censoring system. Finally, we conclude with a discussion of future directions and the therapeutic implications of modulating lipid metabolism in antiviral responses.

## T cell-immunometabolism

Accumulating evidence has highlighted the critical role of cellular metabolism in multiple aspects of T-cell biology. Upon TCR activation, transcriptional programs regulating the metabolism of amino acids, sugars, fatty acids, and lipids are reprogrammed to meet the bioenergetic and biosynthetic demands associated with rapid proliferation and increased cell size. A paradigm shift in immunometabolism emphasizes that metabolites serve not only as building blocks for biosynthesis or substrates for energy generation but also as direct or indirect modulators of key signaling pathways in T cells (1). In naïve T cells, a metabolic shift from OXPHOS to glycolysis is essential for the acquisition of effector functions following antigenic stimulation (18). Accordingly, activated T cells become heavily dependent on aerobic glycolysis to supply ATP and generate metabolic intermediates that sustain intracellular metabolism and maintain mitochondrial membrane potential. Aerobic glycolysis, defined as the conversion of glucose to lactate in the presence of sufficient oxygen, yields only two ATP molecules per glucose molecule, in contrast to the approximately 36 ATP molecules generated per glucose *via* OXPHOS. Despite its lower efficiency in ATP production, aerobic glycolysis facilitates rapid energy generation and provides essential metabolic intermediates for the biosynthesis of lipids, proteins, carbohydrates, and nucleic acids.

Numerous studies have demonstrated a strong link between intracellular metabolic reprogramming and the regulation of immune function. In CD4^+^ TCR-associated activation leads to enhanced nutrient uptake and increased metabolic activity, characterized by elevated transport of glucose, amino acids, and fatty acids. The glucose transporter Glut1 plays a critical role in this process; its deficiency impairs glucose uptake and glycolysis, thereby suppressing TCR-induced cell proliferation and survival, and compromising effector T cell differentiation (19, 20). Similarly, the genetic loss of the glutamine transporter ASCT2 disrupts the differentiation of Th1 and Th17 cells while favoring the development of Tregs (21). Moreover, the inhibition of serine metabolism or the enzyme serine hydroxymethyltransferase 1 (SHMT1) also suppresses optimal T cell proliferation (22).

Beyond the regulation of glucose and amino acid metabolism, lipid metabolism has emerged as a key regulator of T cell responses. Following TCR/CD28 stimulation, T cells undergo rapid proliferation and cellular enlargement, accompanied by an increase in fatty acid uptake. CD4^+^ T cells require metabolic reprogramming of fatty acids for early activation, a process orchestrated by signaling pathways, including the mammalian target of rapamycin (mTOR) and peroxisome proliferator-activated receptor gamma (PPARγ). mTOR integrates signaling pathways associated with nutrient levels, energy status, cellular stress responses, and TCR- and growth factor-mediated signaling (23). PPARγ is a critical transcription factor that regulates lipid metabolism by promoting free fatty acid uptake, facilitating triacylglycerol (TAG) accumulation, and controlling lipolysis in adipose tissue and the liver (24–26). TCR-mediated mTOR activation enhances PPARγ-dependent fatty acid uptake. Furthermore, memory CD8 T cells enhance TAG synthesis and use intrinsic lipolysis pathway to support the metabolic programming necessary for memory generation. In CD4^+^ T cells, Acetyl-CoA Carboxylase 1 (ACC1), which acts as a rate-limiting enzyme in fatty acid biAosynthesis, serves as a marker for the memory potential of individual cells. The expression of the gene encoding ACC1, *Acaca*, is inversely correlated with a memory gene signature in individual single cells. Importantly, the deletion or pharmacological inhibition of ACC1 enhances the generation of memory CD4^+^ T cells (11).

The differentiation of effector CD4^+^ Th subsets is tightly regulated by lipid metabolism. Pharmacological inhibition or genetic deletion of ACC1 disrupts the differentiation of pathogenic Th2 and Th17 cells (7, 8, 27). Especially, recent findings have identified 1-oleoyl-lysophosphatidylethanolamine [LPE (1-18:1)] as a physiological ligand for RORγ, a master transcription factor of Th17 cells (28). Furthermore, both murine and human Th17 cells have been shown to rewire sphingolipid metabolism *via* the serin palmitoyltransferase complex (SPTLC) to sustain their function (29–31). Moreover, medium- and long-chain fatty acids are crucial for the differentiation of Th1 and Th17 cells, while short-chain fatty acids promote the differentiation of Tregs (32). It has been reported that the homeostatic maintenance and suppressive function of lung Tregs rely on FAO and OXPHOS, which are regulated in part by *Acsbg1*-dependent mitochondrial fitness (9).

## Effects of IFN-I on cellular metabolism

**Figure 1:**
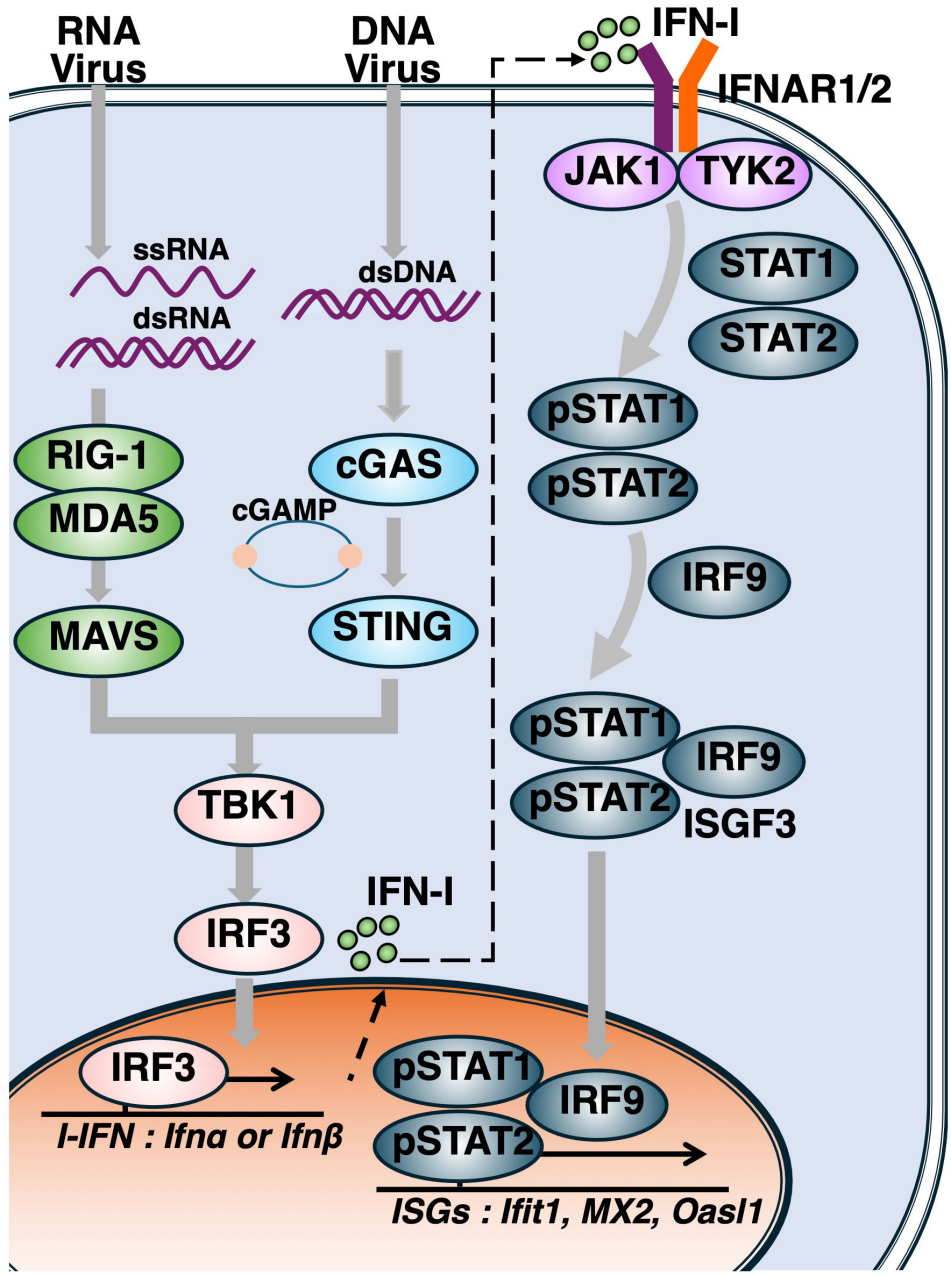
Activation of the cytosolic nucleotide sensing pathways and induction of type I interferon responses. Following viral entry, genomic nucleic acids such as single-stranded RNA (ssRNA), double-stranded RNA (dsRNA), and double-stranded DNA (dsDNA) are released into the cytoplasm. RIG-I and MDA5 detect ssRNA and dsRNA, leading to activation of the adaptor protein MAVS. Cytosolic DNA is recognized by cGAS, which synthesizes cyclic GMP–AMP (cGAMP), a second messenger that activates STING. Upon activation of either MAVS or STING, TBK1 phosphorylates IRF3, initiating the production of type I interferons (IFN-Is). Secreted IFN-Is bind to the type I interferon receptor (IFNAR), activating the JAK–STAT signaling cascade. Phosphorylated STAT1 and STAT2 form a heterodimer that complexes with IRF9 to assemble the ISGF3 complex, which translocates into the nucleus to drive the transcription of interferon-stimulated genes (ISGs).

Upon viral infection, the innate and adaptive immune systems act in concert to eliminate the pathogen and facilitate tissue repair (33). The production and signaling of IFN-I are tightly regulated by the IRF family, which consists of nine members, among which IRF3 and IRF7 serve as critical mediators of IFN-I production. Following the engagement of IFN-I with the IFN-I receptor (IFNAR), the JAK/STAT signaling pathway is activated, leading to the formation of the ISGF3 transcriptional complex, composed of STAT1, STAT2 and IRF9, which drives the expression of ISGs (34) (**Figure 1**).

**Figure 2:**
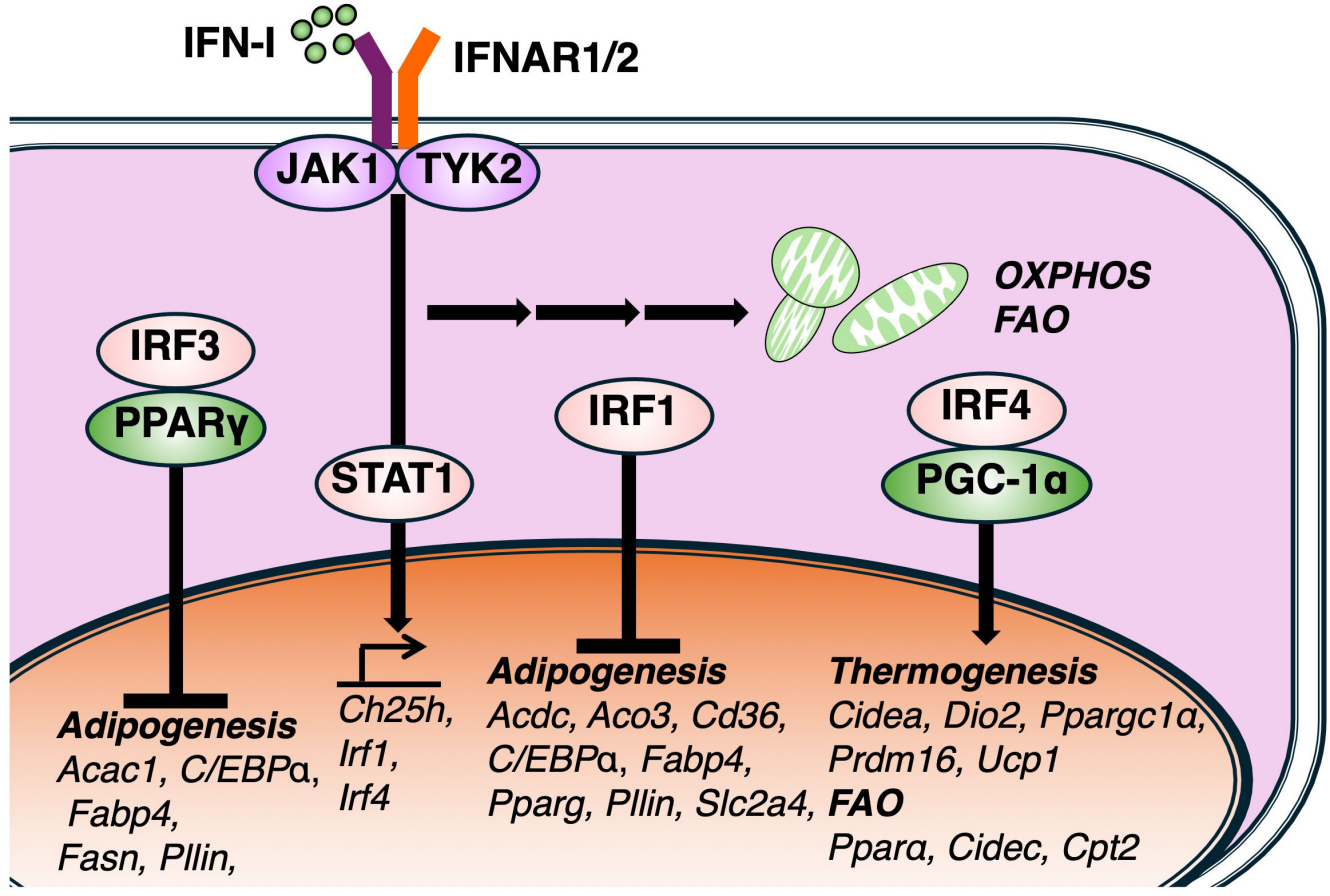
Effects of IFN-I signaling on lipid metabolic pathway. IFN-I signaling regulates lipid metabolic pathway via multiple members of the interferon regulatory factor (IRF) family. IRF3, a key regulator of IFN-I production, negatively regulates the expression of PPARγ and associated lipogenic genes, including, *Acaca, Fabp4, Fas, Plin1*, and its deficiency enhances adipocyte differentiation and lipid accumulation. Other IFN-I-inducible IRFs, such as IRF4, also modulate lipid metabolism genes related to thermogenesis and fatty acid oxidation (FAO). IRF1 also negatively contributes the transcription of adipogenesis genes as well as IRF3. Together, these findings suggest that IFN-I signaling governs lipid metabolism via diverse IRF-dependent transcriptional pathways.

In addition to inducing the expression of ISGs, IFN-I signaling has been shown to modulate lipid metabolism to support essential immune functions. Notably, since the IFNAR is a ubiquitously expressed protein, various types of immune cells respond to IFN-I signaling, thereby regulating both ISG expression and a broad array of metabolic genes (33, 35). These metabolic reprogramming events include alterations in glucose and amino acid metabolism, as well as changes in substrates of the tricarboxylic acid (TCA) cycle; however, changes in lipid metabolism have received particular attention (15, 33, 36, 37). In macrophages, treatment with IFNβ reduces the total amount of cellular cholesterol while simultaneously increasing the production of the antiviral lipid 25-HC. This lipid alters membrane cholesterol accessibility, thereby restricting the replication, entry, and propagation of a broad range of viruses, including murine cytomegalovirus (MCMV), herpes simplex virus type 1 (HSV-1), varicella-zoster virus (VZV), murine gammaherpesvirus 68 (MHV-68) (12, 38, 39). The expression of *CH25H*, which encodes cholesterol 25-hydroxylase and catalyzes the oxidation of cholesterol to 25-HC, is upregulated in macrophages and dendritic cells (DCs) in response to various Toll-like receptor (TLR) agonists and IFN-I signaling (**Figure 2**). IFN-I also rewires the transcriptional network that governs lipid biosynthesis and import in macrophages. Furthermore, administration of CpG or infection with lymphocytic choriomeningitis virus (LCMV) has been shown to elevate OXPHOS and FAO in pDCs, both of which are required for full activation. These metabolic changes are attenuated in *Ifnar1* ^-/-^ pDCs, indicating the role of IFN-I signaling in coordinating cellular metabolic adaptation. In both mouse and human CD4^+^ T cells, IFN-I stimulation also suppresses the expression of genes involved in lipid metabolism, including *Acaca, Acsl3, Fasn, Fads2, Cpt1a, Hmgcs, Hmgcr*, and *Scd2*, further illustrating the role of IFN-I in shaping the immunometabolic landscape.

While IFN-I modulates cellular metabolism and enhances the expression of ISGs, the mechanisms underlying its metabolic regulation remain incompletely understood. Insights from adipocyte biology suggest that members of the IRF family may contribute to metabolic regulation. Several studies have demonstrated that the genetic deletion of specific IRF family members disrupts adipocyte maturation. For instance, a deficiency in IRF3, which is responsible for IFN-I production, leads to impaired glucose homeostasis and promotes the development of type 2 diabetes. IRF3 also negatively regulates the expression of PPARγ, and its deficiency leads to the upregulation of the transcriptional network involved in adipocyte differentiation, including lipogenic genes such as *Acaca*, *Fabp4*, *Fas*, and *Plin1* (40) (**Figure 2**). Supporting this study, another report demonstrated that all nine IRF isoforms are expressed in murine adipose tissue and exhibit dynamic expression profiles during the adipogenesis of *in vitro* generated adipocytes (41). The over expression of IRF3 has a significant effect on lipid accumulation and terminal gene expression such as *Pparγ* and *Cebpa*. In this study, authors have also showed that IRF1 and IRF3 knockdown provoke accumulation of lipid droplet. These studies suggest that IRF3 can regulate the transcription of genes related to lipid metabolism as well as the production of I-IFN. However, further studies are needed to determine whether IRF3 directly binds to the transcriptional regulatory regions of these genes. Notably, while IFN-I signaling is mainly regulated by IRF3, IRF7 and IRF9, other research groups also show additional IFN-I-inducible IRF family members, such as IRF1 and IRF4, have been implicated in the regulation of adipocyte gene. IRF4 is upregulated by cold and cAMP in both mouse and human brown adipocytes. It promotes thermogenesis and resistance to obesity, as shown by overexpression studies. Conversely, IRF4 deficiency impairs thermogenic capacity, leading to obesity and cold intolerance. Mechanistically, IRF4 interacts with PGC-1α to drive thermogenic gene expression (42). Fat-specific IRF4-deficient mice fed a high-fat diet also exhibit increased body weight, highlighting its physiological relevance. Similarly, IRF1 expression is elevated in subcutaneous adipose tissue of obese individuals compared to lean participants (43). Collectively, these findings indicate that IFN-I signaling regulates lipid metabolism through multiple IRF-dependent regulatory pathways across various types of cell development and function.

## Immunometabolism regulates the defense against viral pathogen

**Figure 3:**
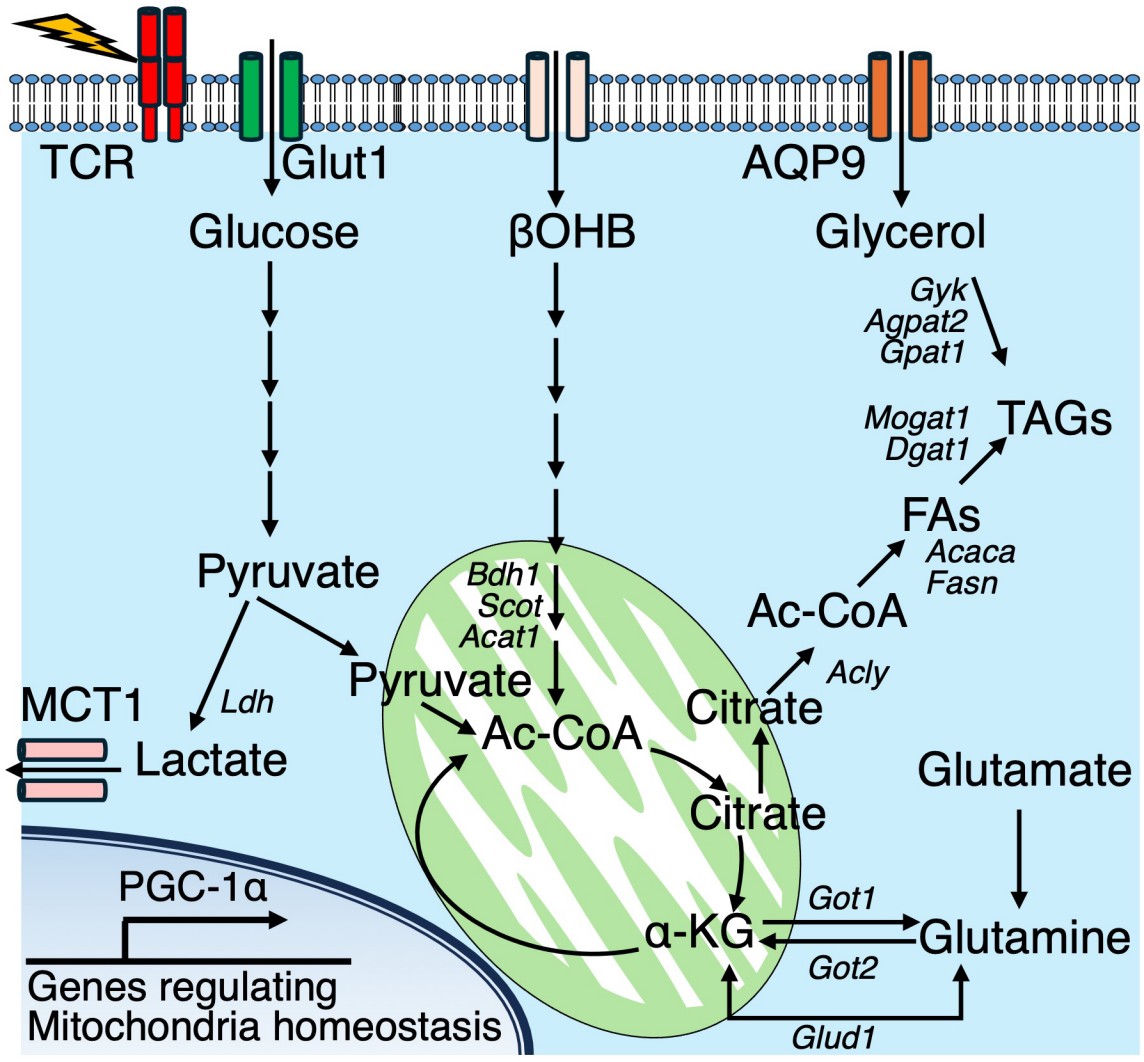
In effector T cells, glucose and lipid metabolic pathways converge to support cellular energy and lipid biosynthesis. TCR signaling activate multiple metabolic pathways. Glucose is metabolized to pyruvate, which is either exported via monocarboxylate transporter 1 (MCT1) or enters the mitochondrial tricarboxylic acid (TCA) cycle. Glutaminolysis contributes α-ketoglutarate (αKG) to the TCA cycle, ultimately supporting the generation of citrate. Cytosolic citrate is converted into acetyl-CoA (Ac-CoA), serving as a precursor for fatty acid synthesis. Along with glycerol, imported via aquaporin 9 (AQP9), Ac-CoA supports triacylglycerol (TAG) biosynthesis. Additionally, the ketone body β-hydroxybutyrate (βOHB) is imported and converted into Ac-CoA, which is further utilized in the TCA cycle. In addition, the ketone body βOHB is taken up from the extracellular space and metabolized into acetyl-CoA, which then enters the mitochondrial TCA cycle.

Increasing evidence highlights the significance of immunometabolism in the context of viral infections. In particular, numerous studies have demonstrated that CD8^+^ T cells dynamically regulate their metabolic programs during LCMV infection. Notably, the metabolic alterations that occur following LCMV infection are largely mediated through mitochondrial pathways, including OXPHOS, FAO, and glutaminolysis. Peroxisome proliferator-activated receptor gamma coactivator 1-alpha (PGC-1α) is a key regulator of mitochondrial biogenesis and modulates both OXPHOS and FAO. Enforced expression of PGC-1α promotes the persistence of CD8^+^ T cells, their memory phenotype, and their antigen recall potential in response to LCMV (44). Glutaminolysis is a metabolic process that converts glutamine into glutamate and subsequently into α-ketoglutarate (α-KG). This process can replenish intermediates in the TCA cycle and fuel OXPHOS. α-KG can be generated either *via* the transamination mediated by glutamic oxaloacetic transaminases (GOT1 and GOT2) or through oxidative deamination by glutamate dehydrogenase 1 (GLUD1). During LCMV infection, effector CD8^+^ T cells predominantly utilize the GOT pathway, while memory CD8^+^ T cells preferentially express GLUD1 (45). GOT1 enhances the function and proliferation of effector CD8^+^ T cells in a manner dependent on Hif-1α and Myc, which are crucial factors to promote glycolysis. In contrast, these same transcription factors have been shown to negatively affect the generation of memory CD8^+^ T cells. In murine models, the expression of the glycerol channel aquaporin 9 (AQP9) is upregulated in CD8^+^ T cells during memory formation (26). AQP deficiency impairs glycerol uptake and subsequent TAG synthesis, thereby compromising the generation of memory T cells. IL-7 stimulation of memory CD8^+^ T cells enhances the mRNA expression of *Aqp9* and genes involved in TAG synthesis, including *Gyk, Agpat2, Gpat1, Mogat1*, and *Dgat1*, alongside the accumulation of lipid droplets (**Figure 3**).

Fatty acid biosynthesis occurs in the cytoplasm using acetyl-CoA, catalyzed by ACC1, while mitochondrial acetyl-CoA is typically oxidized into TCA cycle intermediates. Ketone bodies (KBs), including β-hydroxybutyrate (βOHB) and acetoacetate, serve as alternative carbon sources and are metabolized to acetyl-CoA through a series of enzymatic reactions collectively known as ketolysis. During both acute LCMV Armstrong and chronic LCMV Clone-13 infections, CD8^+^ T cells highly upregulate a set of genes associated with ketone body metabolism (46). Cellular βOHB is catabolized into acetyl-CoA, which serves not only as a metabolic substrate but also as a donor for histone acetylation, subsequently enhancing CD8^+^ T cell effector functions both *in vitro* and *in vivo*. Importantly, metabolic tracing with ^13^C-based substrates revealed that a substantial portion of intracellular βOHB originates from the extracellular environment in CD8^+^ T cells. In co-labeling experiments using [U-^13^C_6_]-glucose and [2,4-^13^C_2_]-βOHB, βOHB contributed approximately 50% more carbon to TCA cycle intermediates compared to glucose. Furthermore, ^13^C from [U-^13^C_4_]-βOHB was detected at histone H3K27, further supporting the role of βOHB-derived acetyl-CoA in regulating epigenetic modifications. These findings identify βOHB as a critical substrate for both metabolic and epigenetic regulation of CD8^+^ T cell effector responses.

**Figure 4:**
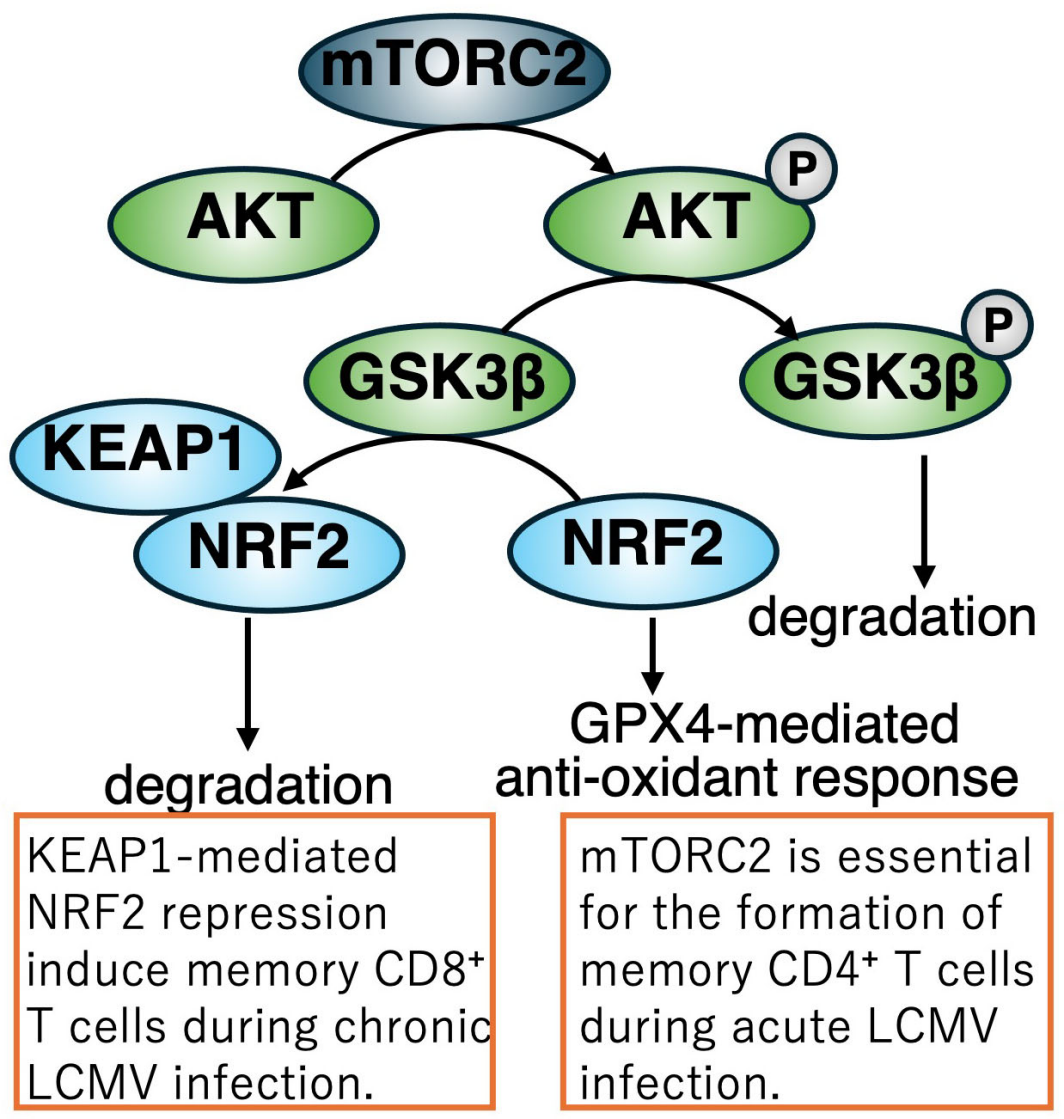
Regulation of memory T cell differentiation during LCMV infection via the mTORC2-NRF2 axis. During acute LCMV Armstrong infection, activation of the mTORC2–AKT signaling pathway in CD4^+^ T cells inhibits GSK3β-mediated degradation of NRF2. As a result, NRF2 is stabilized and induces the expression of GPX4, a key antioxidant enzyme that supports memory CD4^+^ T cell development. In contrast, during chronic LCMV infection, KEAP1 facilitates the proteasomal degradation of NRF2 in CD8^+^ T cells, a process essential for proper memory differentiation and immune homeostasis under persistent viral challenge. Disruption of mTORC2 signaling leads to ferroptosis of memory CD4^+^ T cells due to impaired AKT/GSK3β phosphorylation, mitochondrial ROS accumulation, lipid peroxidation, and suppressed GPX4 expression.

In addition to these metabolic observations, mTORC2 signaling has been implicated in the formation of LCMV-specific memory CD4^+^ T cells (47). Following LCMV infection, SMARTA CD4^+^ T cells, which specifically recognize the LCMV glycoprotein 61-80 epitope, exhibit robust phosphorylation of AKT, a major downstream target of mTORC2. Elevated levels of phosphorylated AKT persist into the memory phase. Furthermore, disruption of mTORC2 signaling during the memory phase leads to a profound loss of virus-specific memory CD4^+^ T cells due to ferroptosis. Mechanistically, mTORC2 inactivation results in impaired phosphorylation of AKT and GSK3β, leading to excessive accumulation of mitochondrial reactive oxygen species (ROS) and subsequent lipid peroxidation, which are hallmarks of ferroptotic cell death. The abrogation of this signaling pathway also suppresses glutathione peroxidase 4 (GPX4), a major scavenger enzyme that detoxifies lipid peroxidation (**Figure 4**).

Activated T cells, upon stimulation through antigen recognition, are essential for managing the byproducts generated by increased bioenergetic activity. Monocarboxylate transporter 1 (MCT1), encoded by *Slc16a1*, facilitates the export of lactic acid, the terminal product of glycolysis. MCT1 deficiency impairs the proliferation of both CD4^+^ and CD8^+^ T cells, and T cell–specific deletion of MCT1/*Slc16a1* renders mice more susceptible to infections by pneumonia virus (PV) and MHV-68 (48) (**Figure 3**). In accordance with TCR-mediated activation, T cells are required to counteract oxidative stress to maintain functional integrity. Kelch-like ECH-associated protein 1 (KEAP1) regulates the activity of NF-E2-related factor 2 (Nrf2), a master regulator of cellular antioxidant responses. Both CD4^+^ and CD8^+^ T cells require KEAP1 for optimal responses during chronic LCMV infection (49) (**Figure 4**). Viral infections broadly reprogram cellular metabolism, sometimes provoke T cell dysfunction. Indeed, hepatitis C virus (HCV) infection induces CD8^+^ T cell exhaustion during the progression from acute to chronic infection, primarily *via* dysregulated glucose and mitochondrial metabolism (50). Of particular note, pharmacological modulation using histone methyltransferase inhibitors and p53 antagonists to target these metabolic alterations has been shown to improve antiviral function and metabolic fitness in exhausted HCV-specific CD8^+^ T cells.

## Pathogen nucleotide sensor

Effective host defense against pathogens is initiated by the detection of nucleic acids through cellular nucleic acid sensing pathways, leading to the upregulation of antiviral ISGs. These sensing mechanisms are integral components of the innate immune system and rely on pattern recognition receptors (PRRs) to detect pathogen-associated molecular patterns (PAMPs), including RNA and DNA (33, 36). Two key cytosolic sensors in this context are mitochondrial antiviral signaling protein (MAVS) and stimulator of interferon genes (STING), which are essential mediators of RNA and DNA sensing pathways, respectively (33, 36). Viral double-stranded RNA (dsRNA) is recognized by the RNA helicases RIG-I and MDA5, which initiate downstream signaling through the activation of MAVS. In parallel, cytosolic viral double-stranded DNA (dsDNA) is sensed by cyclic GMP-AMP synthase (cGAS), which catalyzes the production of cGAMP, a second messenger that activates STING (33, 36). Upon activation, both MAVS and STING stimulate TANK-binding kinase 1 (TBK1), which subsequently phosphorylates and activates IRF3 to induce the production of IFN-I. Interestingly, several recent studies have highlighted a reciprocal relationship between these nucleic acid sensing pathways and cellular metabolism in immune cells, suggesting that metabolic state can influence antiviral immune activation and *vice versa* (**Table 1**).

**Table 1:** List of the relatioinship between MAVA/STING and metabolism.

STING			
Regulatory pathways	Related mehacnism	Effects	Reference
STING modifications	Nitro-fatty acids	Nitro-fatty acids inhibit STING palmitoylation and consequently attenuate IFN-I responses.	(61)
	Palmytoylation	Cys88 and Cys91 of STING are palmitoylated at the Golgi, and are required for the induction of IFN-I responses.	(59)
	Polyunsaturated fatty acid (PUFA)	PUFA inhibits the interaction between STING and its ligand.	(65)
Ligand regulation	Monounsaturated fatty acid (MUFA)	Defects in MUFA biosynthesis lead to the cytosolic DNA accumulation, which is converted into cGAMP, and subsequently activates STING.	(64)
	SMPDL3A	SMPDL3A, induced by LXR activation, cleaves cGAMP into pGpA, thereby limiting STING signaling.	(66)
Localization	Cholesterol	Cholesterol depletion at ER membrane facilitate STING/TBK1 interactions independently of detectable cGAMP.	(16)
	Lipid composition at Goldi	Palmitoylated STING forms oligomeric clusters, each consisting of approximately 20 molecules, at the trans-Golgi network.	(60)
	NPC1	The lysosomal cholesterol transporter NPC1 functions as a cofactor in STING degradation by trafficking STING to lysosomes.	(63)
MAVS			
Regulatory pathways	Related mehacnism	Effects	Reference
Protein interaction	G6D	Upon translocation to peroxisomes, MAVS recruits G6PD to activate the PPP, leading to TRAF6–IRF1–mediated type III IFN production.	(18)
	GFPT2	MAVS associates with GFPT2, TRAF2, and TRAF6 on the mitochondria associated membrane, resulting in the activation of type I IFN.	(18)
MAVS moidfication	O-GlcNacylation	OGT-mediated O-GlcNAcylation at Ser366, using UDP-GlcNAc from the HBP, promotes K63-linked ubiquitination and sustains MAVS activation.	(54)
	Palmytolation	ZDHHC24 palmitoylates MAVS to enhance TBK1–IRF3–IFN signaling, while APT2 removes the modification through MAVS de-palmitoylation.	(55)

**Figure 5:**
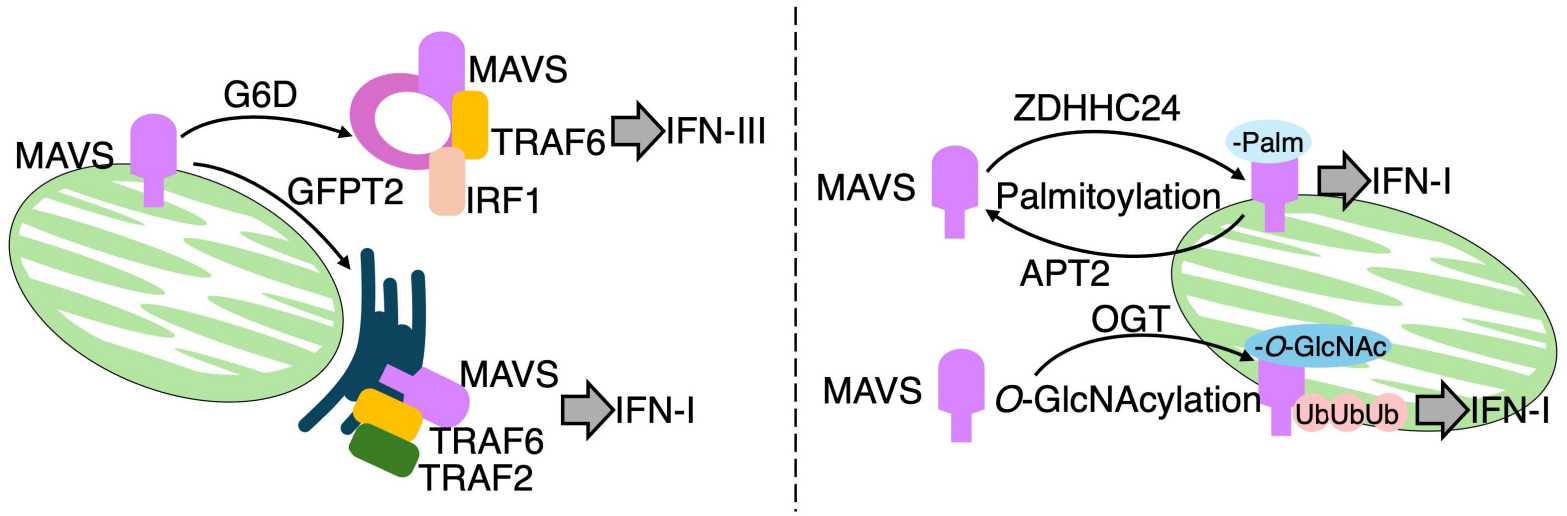
MAVS regulates metabolic reprogramming and immune signaling in response to RNA virus infection. Left panel: RIG-I and MDA5 detect viral ssRNA or dsRNA and activate mitochondrial antiviral-signaling protein (MAVS). MAVS localized to peroxisomes induces a metabolic shift toward the pentose phosphate pathway (PPP), promoting IFN-λ (type III interferon) production. In contrast, MAVS at mitochondria-associated ER membranes (MAMs) activates the hexosamine biosynthetic pathway (HBP), enhancing IFN-I responses. Peroxisomal MAVS interacts with glucose-6-phosphate dehydrogenase (G6PD) and recruits TRAF6 and IRF1 to form a signaling complex. MAM-localized MAVS engages GFPT2 and assembles a complex involving TRAF6 and TRAF2. Right panel: MAVS signaling is modulated by post-translational modifications. Palmitoylation by ZDHHC24 promotes MAVS activation, whereas APT2-mediated depalmitoylation acts as a negative regulator. Additionally, O-GlcNAcylation at serine 366 by O-GlcNAc transferase (OGT), utilizing UDP-GlcNAc from the HBP, promotes K63-linked ubiquitination and sustains MAVS activation.

During Vesicular stomatitis virus (VSV) infection, bone marrow derived macrophages (BMDMs) rewire glucose flux from glycolysis toward the pentose phosphate pathway (PPP), and the hexosamine biosynthesis pathway (HBP), in a manner dependent on MAVS (51) (**Figure 5**: left pannel). A ¹³C_6_-glucose tracing assay clearly revealed substantial incorporation of ¹³C_6_-glucose into PPP intermediates, including 6-phosphogluconate and sedoheptulose-7-phosphate, as well as into uridine diphosphate N-acetylglucosamine (UDP-GlcNAc), a key metabolite of the HBP. In contrast, lower ¹³C_6_ incorporation is observed in lactate, the end product of glycolysis, and in TCA cycle intermediates such as citrate, α-ketoglutarate, and succinate. Notably, pharmacological inhibition of either the PPP or HBP impaired survival in mice following VSV infection. Subcellular localization of MAVS appears to direct the specific routing of glucose flux. Indeed, peroxisomal MAVS is shown to preferentially promote PPP activity and the expression of type III IFN, whereas mitochondria-associated membrane-localized MAVS facilitates HBP activation and the induction of IFN-I. In addition to spatial control, MAVS is regulated through multiple post-translational modifications (**Figure 5**: right pannel). For example, MAVS is O-GlcNAcylated at several sites *via* O-GlcNAc transferase (OGT), and this modification at serine 366 promotes K63-linked ubiquitination, which is critical for MAVS-mediated antiviral signaling both *in vitro* and *in vivo* (52, 53). Further regulation of MAVS activity occurs *via* lipid modifications. A study published in 2024 demonstrated that palmitic acid induces MAVS palmitoylation, aggregation, and activation during VSV and Sendai virus (SeV) infections. Among the 24 members of the S-palmitoyl transferase ZDHHC family, ZDHHC24 is identified as the enzyme responsible for catalyzing MAVS palmitoylation and the subsequent IFN-I response. Conversely, APT2, an acyl-protein thioesterase, is shown to depalmitoylate MAVS, thereby reversing its palmitoylation (54). These findings collectively highlight a complex interplay between MAVS localization, glycosylation, lipidation, and metabolic reprogramming in shaping antiviral immune responses.

**Figure 6:**
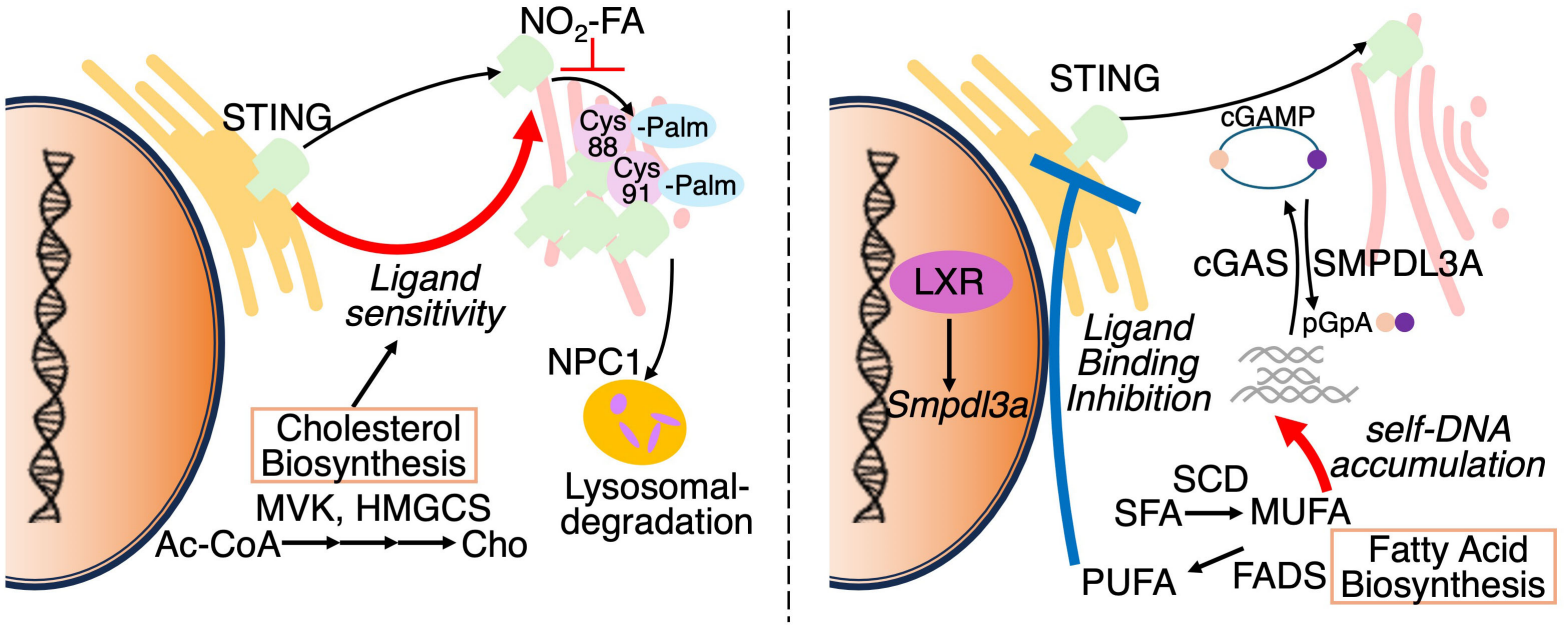
Reguation of STING activation by subcellular localization and lipid metabolism. Left panel: STING resides in the ER under basal conditions and translocates to the Golgi upon cGAMP binding. At the Golgi, STING undergoes palmitoylation and forms oligomeric clusters within cholesterol-rich microdomains to initiate antiviral signaling. Palmitoylation is inhibited by nitro-fatty acids (NO_2_-FAs). NPC1 mediates STING degradation via lysosomal trafficking. Altered cholesterol metabolism modulates ER membrane composition, sensitizing STING to activation. Right panel: cGAS synthesizes cGAMP from cytosolic DNA to activate STING. Conversely, SMPDL3A degrades cGAMP into pGpA, limiting STING signaling. Suppression of monounsaturated fatty acid (MUFA) synthesis enhances genomic DNA–derived cGAMP production. Polyunsaturated fatty acids (PUFAs) interact with STING and suppress its activity, indicating lipid-dependent regulation of the cGAS–STING axis.

Among various metabolic pathways, lipid metabolism has emerged as key modulator of STING activity. Under steady state conditions, STING resides in the ER and translocate to perinuclear compartments including the Golgi apparatus, upon ligand recognition (55, 56) (**Figure 6**: left pannel). Mukai et al. have also demonstrated that Cys88 and Cys91 of STING are palmitoylated at the Golgi, and required for the induction of IFN-I responses (57). Notably, palmitoylation of STING is essential for the formation of oligomeric clusters, each consisting of approximately 20 molecules, at the trans-Golgi network (TGN). Disruption of cholesterol trafficking to the TGN has been shown to impair cluster formation, indicating that this process is critically dependent on the local lipid microenvironment. In particular, cholesterol is thought to contribute to the assembly of lipid raft-like domains within the Goldi, thereby promoting oligomerization of STING (58). Importantly, inhibition of STING palmitoylation suppresses IFN-I responses triggered by pathogenic STING variants associated with STING-associated vasculopathy with onset in infancy (SAVI), an autoinflammatory disease characterized by constitutive activation of the STING pathway. Furthermore, nitro-fatty acids generated during HSV-2 infection have been shown to inhibit STING palmitoylation and consequently attenuate IFN-I responses in fibroblasts derived from SAVI patients. These findings highlight STING palmitoylation as a potential therapeutic target in STING-driven autoinflammatory disorders (59).

Cholesterol metabolism has also been shown to regulate STING signaling. In macrophages, limitation of cellular cholesterol, specifically through genetic deletion of Srebf2, which encodes sterol regulatory element-binding protein 2 (SREBP2), causes spontaneous activation of STING-IRF3 axis and elevated IFN-I responses (14, 38, 60) (**Figure 6**: left pannel). Interestingly, this response is diminished by the deletion of *Mb21d1*, which encodes the cGAS protein, despite undetectable cGAMP levels (~240 femtomoles) in both control and *Srebf2*-deficient cells, suggesting that ER membrane cholesterol depletion facilitate STING/TBK1 interactions independently of detectable cGAMP. Supporting this, another group has shown that cGAMP stimulation leads to a transient decline in ER cholesterol levels in a SOAT1 dependent manner (61). In addition, the lysosomal cholesterol transporter Niemann–Pick type C1 (NPC1) functions as a cofactor in STING degradation by trafficking STING to lysosomes both in human and murine cells (62). Notably, genetic deletion of *Npc1* leads to STING accumulation and its tethering to SREBP2, thereby priming STING activation. Thus, these findings highlight that alterations in the lipid composition or trafficking pathways can profoundly modulate STING.

In contrast to macrophages, T cells engage the cGAS-STING pathway under conditions of fatty acid metabolic stress. In fact, suppression of fatty acid metabolism enables T cells to autonomously produce IFN-I *via* STING activation (**Figure 6**: right pannel. Our recent study combining CRISPR-based gene edition with non-targeting lipidomics revealed that suppression of SCD2, an enzyme responsible for MUFA synthesis, elicits STING-dependent antiviral responses (14). In this study, *Scd2*-deficient Th1 cell accumulated cGAMP and exhibited elevated levels of cytosolic genomic DNA, rather than mitochondrial DNA, suggesting that nuclear genome derived self-DNA trigged cGAS-STING activation in this context. Following STING activation, IRF3, but not IRF7, is responsible for the IFN-I production in *Scd2*-defects Th1 cell (63). Furthermore, I-IFN stimulation induces IRF9 to directly bind to the transcription sites of a wide range of ISGs. Although genetic deletion of *Fads2*, which is desaturase of polyunsaturated fatty acids (PUFAs), had minimal effects on the induction of ISGs, another recent study has shown that activation of STING upregulates FADS2, and the resulting PUFAs can inhibit STING signaling, suggesting a negative feedback loop (64). Additionally, the liver X receptor (LXR), a nuclear receptor that governs lipid metabolism, has been shown to attenuate 2′3′-cGAMP-mediated immune responses. Mechanistically, LXR activation induces the expression of sphingomyelin phosphodiesterase acid-like 3A (SMPDL3A), an enzyme that selectively cleaves 2′3′-cGAMP, thereby attenuating cGAS–STING signaling (65). Taken together, these findings illustrate that lipid metabolism modulates IFN-I responses through the regulation of STING ligands such as cGAMP.

## Alteration of lipid metabolism during viral infection

Host cells enhance antiviral response by rewiring cellular lipid metabolism. However, viruses, which lack the intrinsic capacity for lipid biosynthesis, hijack host lipid metabolic pathways to support their replication and assembly. Furthermore, during viral infection, specific lipid species are generated that can either inhibit or enhance antiviral responses, rendering lipid metabolism a double-edged sword for the host during viral infection (**Table 2**).

**Table 2:** List of the relatioinship between virus replication and lipid.

Virus species	Lipid-related change	Effect on viral replication	Reference
HCMV (Human cytomegalovirus)	HCMV promotes the elongation of pre-existing fatty acids through ELOVL7, leading to the production of VLCFA, and increases fatty acid saturation in host cells.	Enhances viral envelope formation and replication. ELOVL7 deficiency impairs replication.	(67)
HAV (Hepatitis A virus)	HAV reprograms lipid biosynthetic pathways by upregulating Acsl4, Elovl4, Elovl7, and Slc27a2, resulting in the production of VLCFA-containing phospholipids.	Supports viral RNA replication	(68)
HAV (extracellular quasi-enveloped)	The quasi-enveloped form of HAV virions is enriched in sphingolipids, particularly ceramides containing VLCFA tails.	Essential for virion structure and stability	(68)
RV (Rotavirus)	RV infection increases the abundance of various ceramides in host cells.	C16–C24 ceramide treatment inhibits replication	(69)
IAV (Influenza A virus)	IAV activates the SREBP2 pathway via STAT3 signaling, leading to increased cholesterol biosynthesis.	Enhances replication; STAT3 inhibition suppresses SREBP2 and reduces viral propagation.	(70)
ZIKV (Zika virus)	ZIKV increases the expression of SREBP2 and ABCG1, altering cholesterol metabolism and lipid droplet dynamics.	SREBP2 inhibition suppresses ZIKV; ABCG1 deficiency increases LDs and enhances replication	(71)
SARS-CoV-2	SARS-CoV-2 hijacks host lipid droplet formation pathways involving ACAT and DGAT1.	Inhibition of ACAT or DGAT1 suppresses replication	(72)
HCV (Hepatitis C virus)	HCV stimulates lipid droplet formation by enhancing ACAT and DGAT1 activity, resulting in cholesterol ester and TAG accumulation.	ACAT or DGAT1 inhibition reduces replication	(73)

**Figure 7:**
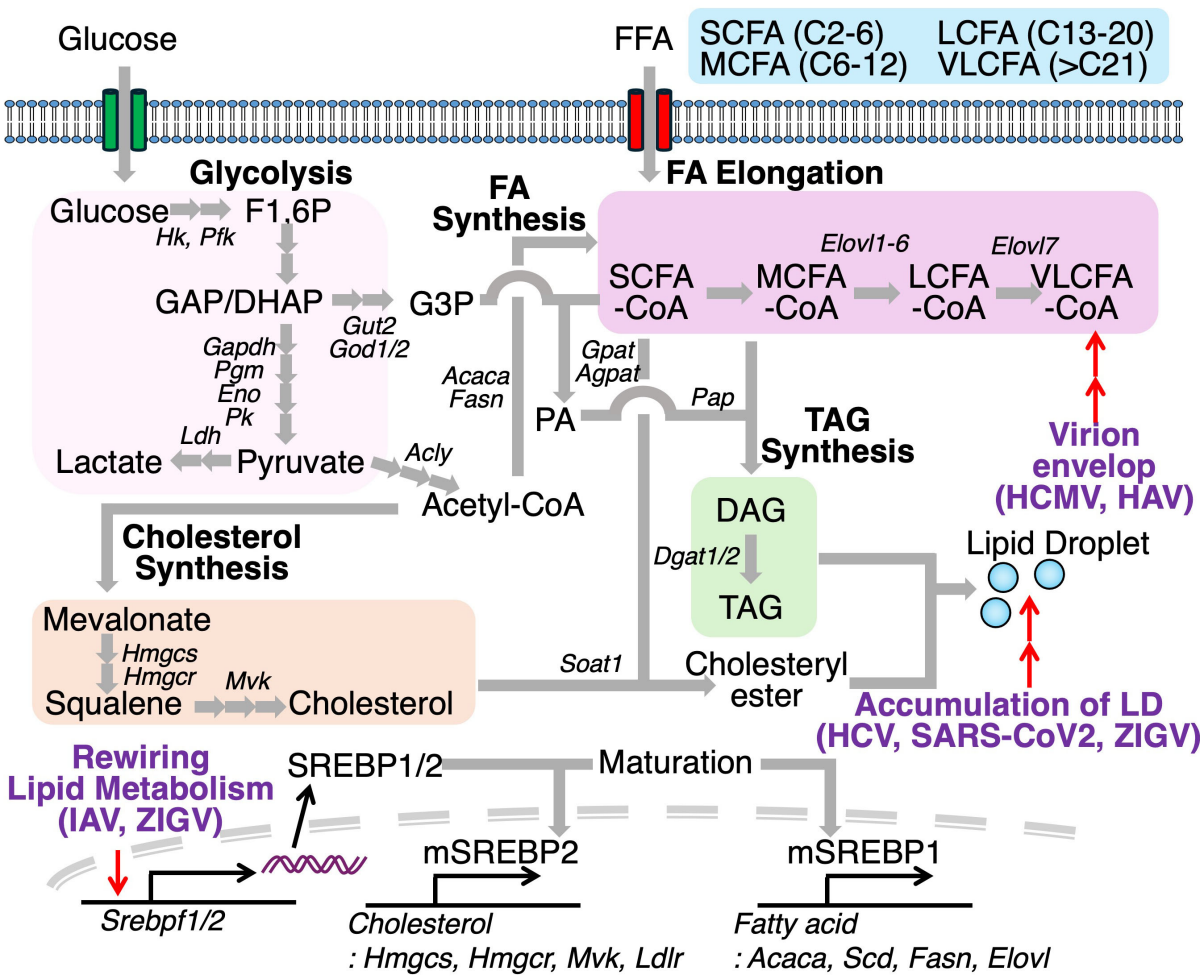
Viral exploitation of host lipid metabolism facilitates replication and pathogenesis. Viruses reprogram host lipid metabolic pathways to promote replication. During human cytomegalovirus (HCMV) or hepatitis A virus (HAV) infection, fatty acid elongation is enhanced, increasing production of very-long-chain fatty acids (VLCFAs). Infections with influenza A virus (IAV) and Zika virus (ZIKV) upregulate SREBP1 and SREBP2, transcription factors that promote cholesterol and fatty acid biosynthesis, contributing to lipid droplet accumulation. Inhibition of key enzymes in lipid droplet formation, such as DGAT1 and SOAT1, improves antiviral immunity, underscoring the therapeutic potential of targeting host lipid metabolism during infection.

Several viruses manipulate host lipid metabolic pathways to induce the biosynthesis of lipids that are not typically produced under physiological conditions. Human cytomegalovirus (HCMV), for instance, promotes the elongation of pre-exiting fatty acids to produce very long chain fatty acids (VLCFA) (>C21), which are incorporated into the virion envelope (66) (**Figure 7**). HCMV infection also increases the degree of fatty acid saturation in host fibroblasts, with saturated fatty acids (SFAs) comprising over half of the total fatty acid content in HCMV. Furthermore, elongase ELOVL7 protein is markedly upregulated during HCMV infection, despite being undetectable under basal conditions. This study also shows that overexpression of ELOVL7 enhances viral replication, whereas its deletion attenuates viral infection. A similar increase in VLCFA production has been observed in hepatitis A virus (HAV)-infected cells (67). HAV infection reprograms a lipid biosynthetic network, including *Acsl4, Elovl4, Elovl7*, and *Slc27a2*, which promotes the synthesis of phospholipids containing VLCFA tails, essential for viral RNA replication. Furthermore, extracellular quasi-enveloped HAV virions are also enriched in sphingolipids, including VLCFAs-containing ceramides (Cer). Another study has investigated the lipid composition of rotavirus (RV), which possesses a genome of 11 double-stranded RNA segments (68). RV-infected cells elevate the amounts of various kinds of Cer. Treatment of C16-, C18-, C24-Cer hinder RV propagation at the replication step. In addition to fatty acid remodeling, viral infection is also known to activate sterol metabolic pathway. Indeed, IAV infection induces activation of sterol response element binding protein 2 (SREBP2) in NL20 cells, a noncancerous human bronchial epithelial cell line, and 293T cells, a human kidney epithelial-like cell line *via* STAT3 signaling (69). Consistent with this, pharmacological inhibition of STAT3 blocks the activation of SREBP2 and inhibits IAV replications. Similarly, ZIKV infection increases the expression of both SREBP2 and ATP binding cassette transporter G1 (ABCG1), a cholesterol efflux transporter, in retinal pigment epithelial cells (54, 70). Inhibition of SREBP2 reduces cellular cholesterol levels and suppresses ZIKV infection, whereas ABCG1 deficiency enhances lipid droplet accumulation and promotes ZIKV replication, suggesting that ABCG1 limits intracellular cholesterol availability for viral exploitation. LDs, typically abundant in mature adipocytes, also accumulate in virus-infected or activated immune cells due to viral manipulation such as hijacking or cell-intrinsic activation of host lipid metabolism. Acyl-CoA:cholesterol acyltransferase (ACAT, also known as sterol O-acyltransferase, SOAT), catalyzes the esterification of free cholesterol to form cholesterol esters (ChE) stored in LDs along with diacylglycerol and TAG, serving as energy sources. Pharmacological inhibition of ACAT has been shown to reduce replication of hepatitis C virus and SARS-CoV2 (71, 72). Similarly, targeting DGAT1, which catalyzes TAG synthesis, also confers antiviral effects against SARS-CoV-2, HCV, and ZIKV.

Taken together, these findings suggest that targeting host lipid metabolic pathways to limit the availability of lipids required for virus replication may serve as an effective strategy to combat viral infections. Conventional antiviral therapies primarily target viral proteins, but such approaches are frequently compromised by the rapid emergence of drug-resistant variants. In contrast, given that viruses lack endogenous lipid metabolic enzymes, interventions that modulates host lipid metabolism may offer a promising alternative with a potentially lower risk of resistance development. However, the types of altered lipids during infection can vary by virus species, suggesting that the antiviral effects may differ depending on which lipid metabolic enzymes are targeted. A more detailed investigation is needed to understand how lipid composition changes across different virus species.

## Systemic changes in lipid metabolism

Fluctuations in lipid metabolism during pathogen infection have profound impacts on viral replication, inflammatory responses, and dissemination, as well as intrinsic host defense mechanisms. Consequently, host metabolic status not only represents a potential therapeutic target but may also serve as a biomarker for predicting disease outcomes. To this end, virus-induced alterations in genes and substrates of lipid metabolism have been characterized using next-generation sequencing or mass spectrometry approaches.

In murine models of severe influenza virus A (IAV) infection, significant changes in lipid composition have been detected in both plasma and lung tissues (73). Remarkably, alterations in the amounts of PUFA-containing phosphatidylethanolamine (PEs) correlate with infection dose and disease severity. PE (20:4) and PE (22:6) levels are elevated in lungs and plasma, respectively, of IAV-infected mice. Furthermore, IAV-infected lungs increase mRNA expression of *Etnk1* and *Ept1*, the rate-limiting enzymes of *de novo* PE biosynthesis, as well as *Acsl4* and *Pla2g4a*, which are involved in the remodeling of PUFA hydrolyzed from cellular membranes. These findings suggest a coordinated reprogramming of both *de novo* synthetic and remodeling pathways of PE metabolism during infection. A recent study by *Jia* et al., has explored the role of oleoyl-ACP-hydrolase (OLAH), a crucial enzyme in fatty acid synthesis, as an important mediator of disease severity across multiple respiratory virus infections in human cohorts (74). Transcriptomic analyses revealed a strong association between *OLAH* and lethal outcomes of fatal H7N9 IAV infection shortly after hospital admission, with high levels persisting until death. In contrast, patients who recovered maintained low *OLAH* expression throughout their hospitalization. In addition, elevated levels of *OLAH* were observed in patients with life-threatening seasonal influenza, COVID-19, and respiratory syncytial virus (RSV), but not in mild cases. Additionally, oleic acid abundance is significantly higher in hospitalized COVID-19 patients as compared to non-hospitalized individuals. In relation to these evidences, *Olah*-deficient mice exhibited enhanced viral clearance of H3/N2 IAV, associated with the limitation of oleic acid availability and lipid droplet formation in macrophages. Conversely, mice fed a diet enriched in oleic acid (containing 7.7% oleic acid and 5.8% palmitic acid) increased viral replication and greater weight loss, indicating a diet-infection interaction mediated by lipid metabolism.

In the context of chronic viral infection, human immunodeficiency virus (HIV) infection and subsequent treatment with combination antiretroviral therapy (cART) have been associated with perturbation in lipid profiles. It has been observed that there are decrease in the levels of HDL cholesterol and increase in the levels of LDL cholesterol, total cholesterol and TAGs in HIV-infected individuals. These lipid alterations induced by HIV or its related therapies are thought to contribute to the elevated risk of cardiovascular diseases (75). Plasma metabolites in untreated HIV patients appears to be different from those in a non-HIV infected controls population (76), including reduced levels of biogenic amines such as serotonin, O-PE, glutathione, sarcosine, taurine, and tryptophan. Lipidomic profiling has revealed significant reductions in phosphatidylcholine (PC) levels and increases in the levels of lyso phosphatidic acid (PA) and TAG. Furthermore, after 12 months of cART, metabolic reprogramming is evident, with significant increase of tryptophan and histidine levels and widespread increases in lipids such as PC, lysoPC, PE, sphingomyelin (SM) and ChE.

## Nutritional modulation of host responses to viral infections

Since cellular metabolism shapes immune cell activation and function, dietary composition can influence host resistance to virus infection. Indeed, the type of diet affects infection outcomes in animal experimental models. For example, mice fed a grain-based diet successfully recovered from IAV infection, whereas those fed the AIN93G diet, defined as a highly processed diet, failed to recover (77). Intriguingly, AIN93G-fed mice deficient in the IFNγ receptor showed improved survival following IAV infection, suggesting that highlighted susceptibility associated with processed diet is mediated by IFNγ signaling. Furthermore, dietary salt intake also appears to modulate antiviral immune responses. Mice fed with a high-salt diet was found to protect from lethal VSV infection. Consistently, human macrophages exposed to high salt conditions *in vitro* upregulate IFN-I signaling. Micronutrients such as vitamins have also been investigated in the context of viral susceptibility. The administration of vitamin (Vit) C has been shown to reduce ACE2 proteins, which serve as a primary cellular entry receptor for SARS-CoV-2, in both *in vitro* and *in vivo* models, and restricts viral infection (78). However, similar antiviral effects were not observed with *in vitro* treatment of other vitamin family including VitB1, VitB6, VitB12, VitD3, and VitK1. In contrast, another study demonstrated that dietary supplementation with VitD3 at 10 times the standard intake, enhanced resistance to MHV infection by mitigating pulmonary inflammation and acute lung tissue damage, indicating context-dependent roles for vitamins in antiviral responses (79).

In viral infections, disease severity is controlled not only by viral replication but also by immunopathology such as tissue damage resulting from excessive immune activation. Certain dietary components have been shown to limit virus-induced immunopathology. For example, inflammasome activation and neutrophil-mediated cytotoxicity are known to contribute substantially to pulmonary injury during IAV infection. Dietary fibers and their microbial fermentation products, such as short chain fatty acid (SCFAs: acetate, propionate, and butyrate) produced by commensal bacteria, are well documented to influence immune homeostasis and are involved in the regulation of infectious diseases, allergies, and autoimmune disorders (32, 80). Dietary fiber and SCFAs can facilitate immunomodulatory effects on various immune cell types and have been shown to protect against IAV-induced pathology through two distinct mechanisms (80). A high-fiber diet suppresses the production of CXCL1, a neutrophil-recruiting chemokine, by lung monocytes and macrophages, thereby limiting excessive neutrophil infiltration and subsequent tissue damage. In parallel, SCFAs enhance antiviral CD8^+^ T cell responses *via* FFAR3 (also known as GPR41), one of the receptors for SCFAs. In addition to SCFAs, Goldberg et al., have shown that βOHB suppress activation of the NLRP3 inflammasome in neutrophils and macrophages, thereby reducing IL-1β secretion (81). This study subsequently revealed that a ketogenic diet (KD) protects mice from lethal IAV infection and disease. Furthermore, mice fed a KD-diet upregulate mRNA of *Tcrg-C1*, which is a segment of γδTCR, and depletion of γδT cells diminished survival following IAV-infection under KD feeding conditions.

Obesity has been associated with increased morbidity and mortality following viral infections, including those caused by IAV, SRAS-CoV2, and Dengue virus (DENV). Clinical study has demonstrated that obesity alters the airway milieu, leading to impaired immune responses in bronchoalveolar lavage (BAL) cells against clinically relevant influenza virus; A/Eng/195 (pandemic H1N1/09), A/Eng/691/10 (seasonal H3N2) and B/Florida (influenza B) (82). Furthermore, leptin levels are elevated in both the upper and lower airways of obese individuals compared to their non-obese counterparts. In murine models, leptin treatment suppresses antiviral immune responses in the airway against X31 IAV strain and impairs IFN-I responses in *ex vivo*-cultured alveolar macrophages (82). Furthermore, obesity induced by either a high-fat diet or by genetic deletion of the *Lep* gene (encoding leptin) compromises the host immune responses to H1N1 IAV infection (83). In these obese mice, IAV nucleoproteins have been observed in thoracic adipose tissue located in the mediastinum, between the lungs, heart, and pleura. Similarly, in LCMV-infected obese mice, viral replication occurs in white adipose tissue, accompanied by an accumulation of virus-specific T cells (84). Upon re-exposure to the virus, these T cells mediate severe immunopathology, including acute pancreatitis and adipose tissue necrosis, ultimately leading to mortality. Notably, viral accumulation in thoracic adipose tissue has also been reported in COVID-19 (83). These findings emphasize the immunomodulatory role of adipose tissue in obesity and highlight the potential of dietary interventions to fine-tune immune responses, enhancing antiviral defense while minimizing immunopathological damage.

## Concluding remarks and future perspectives

It is now clear that metabolic changes play an important role during viral infection. Accumulating evidence has shown that intracellular metabolism is essential for regulating immune functions to control pathogen invasion. During viral infection, IFN-I response also rewires the transcriptional networks of genes involved in lipid metabolism as well as antiviral response. Importantly, since viruses lack the intrinsic capacity for lipid biosynthesis, they hijack host lipid metabolic pathways to support their replication and assembly. Indeed, virus-infected cells often tend to enhance lipid biosynthesis pathway alongside with accumulation of lipid droplet. Furthermore, targeting lipid droplet biosynthesis repress viral replication. These changes in the lipid metabolism also affect the function of virus sensing pathway, such as cGAS-STING and RIG-I/MDA-5-MAVS axis, due to the generation of these ligands, enhancement of ligand sensitivity, and post-translational regulation. Thus, lipid metabolic pathways are thought to contribute to antiviral responses not only by regulating metabolic molecules, but also by modulating the activity of conventional antiviral factors.

## Glossary

25-HC: 25-hydroxycholesterol
ABCG1: ATP binding cassette transporter G1
ACAT: Acyl-CoA:cholesterol acyltransferase
ACC1: Acetyl-CoA Carboxylase 1
AQP9: Aquaporin 9
BAL: Bronchoalveolar lavage
BMDM: Bone marrow derived macrophage
cART: Combination antiretroviral therapy
cGAS: cyclic GMP-AMP synthase
ChE: Cholesterol esters
DENV: Dengue virus
dsDNA: Double-stranded DNA
dsRNA: Double-stranded RNA
FAO: Fatty acid oxidation
GLUD1: Glutamate dehydrogenase 1
GOT: Glutamic oxaloacetic transaminases
GPX4: Glutathione peroxidase 4
HAV: Hepatitis A virus
HBP: Hexosamine biosynthesis pathway
HCMV: Human cytomegalovirus
HCV: Hepatitis C virus
HIV: Human immunodeficiency virus
HSV: Herpes simplex virus
IAV: Influenza virus A
IFN: Interferons
IRF: Interferon regulatory factor
ISG: Interferon-stimulated gene
KBs: Ketone bodies
KD: Ketogenic diet
KEAP1: Kelch-like ECH-associated protein 1
LCMV: Lymphocytic choriomeningitis virus
LXR: Liver X receptor
MAVS: Mitochondrial antiviral signaling protein
MCMV: Murine cytomegalovirus
MCT1: Monocarboxylate transporter 1
MHV-68: Murine gammaherpesvirus 68
mTOR: Mammalian target of rapamycin
MUFA: Monounsaturated fatty acid
NPC1: Niemann–Pick type C1
Nrf2: NF-E2-related factor 2
OGT: O-GlcNAc transferase
OLAH: Oleoyl-ACP-hydrolase
OXPHOS: Oxidative phosphorylation
PA: Phosphatidic acid
PAMP: Pathogen-associated molecular patterns
PC: Phosphatidylcholine
pDC: Plasmacytoid dendritic cell
PE: Phosphatidylethanolamine
PGC-1α: Peroxisome proliferator-activated receptor gamma coactivator 1-alpha
PPARγ: Peroxisome proliferator-activated receptor gamma
PPP: Pentose phosphate pathway
PRR: Pattern recognition receptors
PUFA: Polyunsaturated fatty acids
PVM: Pneumonia virus of mice
ROS: Reactive oxygen species
RSV: Respiratory syncytial virus
SARS-CoV-2: Severe Acute Respiratory Syndrome Coronavirus 2
SCFA: Short chain fatty acid
SeV: Sendai virus
SHMT1: Serine hydroxymethyltransferase 1
SM: Sphingomyelin
Sptlc: Serin palmitoyltransferase complex
SREBP: Sterol response element binding protein
STING: Stimulator of interferon genes
TAG: Triacylglycerol
TBK1: TANK-binding kinase 1
TCA cycle: Tricarboxylic acid cycle
TCR: T cell receptor
TGN: Trans-Golgi network
Th: Helper T cells
TLR: Toll-like receptor
UDP-GlcNAc: Uridine diphosphate N-acetylglucosamine
Vit: Vitamin
VLCFA: Very long chain fatty acids
VSV: Vesicular stomatitis virus
VZV: Varicella-zoster virus
βOHB: β-hydroxybutyrate.
